# Diagnosing acquired syphilis through oral lesions: the 12 year experience of an Oral Medicine Center^☆^

**DOI:** 10.1016/j.bjorl.2018.12.010

**Published:** 2019-02-23

**Authors:** Michelle Danielle Porto Matias, Alessandro Oliveira de Jesus, Renata Gonçalves Resende, Patrícia Carlos Caldeira, Maria Cássia Ferreira de Aguiar

**Affiliations:** a Universidade Federal de Minas Gerais (UFMG), Faculdade de Odontologia, Departamento de Cirurgia Oral e Patologia Oral, Belo Horizonte, MG, Brazil; b Hospital Metropolitano Odilon Behrens, Medicina Oral, Belo Horizonte, MG, Brazil

**Keywords:** Syphilis infection, Oral lesions, Diagnosis, Epidemiology, Infecção por sífilis, Lesões orais, Diagnóstico, Epidemiologia

## Abstract

**Introduction:**

A resurgence of syphilis in Brazil has been reported in recent years.

**Objective:**

With this in mind, the present study sought to investigate the frequency, demographics, and clinical characteristics of patients with acquired syphilis with oral involvement who received medical care at an Oral Medicine Reference Center in a Brazilian Public Hospital.

**Methods:**

A retrospective study, spanning a period of 12 years, was performed to identify changing trends in syphilis over time. Medical records from all patients diagnosed with acquired syphilis who received medical care at the Hospital's Oral Medicine Clinic from 2005 to 2016 were reviewed, and the demographic and clinical data were collected.

**Results:**

A total of 85 patients had been diagnosed with acquired syphilis, with a significant increase in the number of cases over the past 5 years. Patients ranged from 16 to 76 years of age, with a peak in the third and fourth decades. Forty-eight cases affected males (56.5%), while 37 cases affected females (43.5%). Most of the oral lesions appeared as unique ulcers or plaques, with the lips and tongue representing the most affected sites. All cases were positive for Venereal Disease Research Laboratory or Fluorescent Treponemal Antibody Absorption, and treatment was performed with Penicillin G benzathine in most cases (84.7%).

**Conclusion:**

The frequency of oral syphilis has been rising over time and oral lesions may well represent a diagnostic clue; therefore, oral health professionals must be made aware and properly trained in an attempt to develop a high degree of clinical suspicion in the diagnosis of syphilis.

## Resumo

**Introdução:**

Um ressurgimento da sífilis no Brasil tem sido relatado nos últimos anos.

**Objetivo:**

Investigar frequência, as características demográficas e clínicas dos pacientes com sífilis adquirida com envolvimento oral que receberam atendimento médico em um centro de referência em medicina oral em um hospital público brasileiro.

**Método:**

Estudo retrospectivo, abrangeu 12 anos, feito para identificar tendências de mudança na sífilis. Registros médicos de todos os pacientes diagnosticados com sífilis adquirida que receberam atendimento médico na clínica de medicina oral do hospital de 2005 a 2016 foram revisados e os dados demográficos e clínicos foram coletados.

**Resultados:**

Foram diagnosticas 85 pacientes com sífilis adquirida, com um aumento significativo no número de casos nos últimos 5 anos. A idade dos pacientes variou de 16 a 76 anos, com pico na terceira e quarta décadas. 48 casos eram do sexo masculino (56,5%) e 37 do sexo feminino (43,5%). A maioria das lesões orais apareceu como úlceras ou placas únicas, os lábios e a língua representaram os locais mais afetados. Todos os casos foram positivos para *Venereal Disease Research Laboratory* e *Fluorescent Treponemal Antibody Absorption Test* e o tratamento foi feito com penicilina G benzatina na maioria dos casos (84,7%).

**Conclusão:**

A frequência da sífilis oral tem aumentado com o tempo e as lesões orais podem representar uma pista diagnóstica; portanto, os profissionais de saúde bucal devem ser conscientizados e devidamente treinados na tentativa de desenvolver um alto grau de suspeição clínica no diagnóstico da sífilis.

## Introduction

Syphilis is a sexually transmitted disease caused by the spirochete “*Treponema pallidum*”, including infection through orogenital contact. Another infective route is congenital syphilis, in which the disease is transmitted during pregnancy.^1^ In both forms, whether congenital or acquired, the oral cavity is the most frequent site of the extragenital manifestation of syphilis.^2^ The most affected sites for secondary syphilitic lesions are tongue, gingiva, soft palate, and lips. Oral lesions commonly appear as ulcers and mucous plaques.^3^

In the 1950s, syphilis was eradicated in developed countries, and due to the discovery of penicillin, a significant decline in the incidence of the disease was observed.^4^ However, a resurgence of the disease has been reported in recent years.^5^ Many studies describe this increasing burden in France, the Netherlands, Sweden, Germany, Ireland, Norway, and Great Britain.^6^, ^7^, ^8^, ^9^ These studies support the fact that the increasing number of syphilis cases is associated with high-risk sexual behavior, coinciding with the new era of Human Immunodeficiency Virus (HIV) infection.^10^ In this sense, syphilis continues to be a major public health problem in Brazil. The detection rate of acquired syphilis was 43.7 cases per 100 million habitants in 2015. From 2010 to 2016, 227,663 cases were diagnosed.^11^, ^12^, ^13^ This scenario points to the need for broad informative campaigns, coupled with preventive actions by the government and non-governmental organizations.^11^

The current study sought to describe the frequency, demographics, and clinical characteristics of acquired syphilis diagnosed through oral manifestations at an Oral Medicine reference center at a public hospital. Improvements in knowledge regarding epidemiological and oral manifestations of syphilis are essential in order to guide dentists and health professionals toward a correct and prompt diagnosis and prevention of the disease.

## Methods

The study protocol was approved by the Institutional Committee of Ethics on Research (protocol no. 55609516.1.0000.5149).

A retrospective study was performed at the Oral Medicine Service of the Hospital Metropolitano Odilon Behrens, in the city of Belo Horizonte, Brazil. This hospital is a regional reference center in oral medicine. None of the patients sought medical treatment before being treated at the Oral Medicine Service. The medical records of all patients diagnosed with oral manifestations of acquired syphilis (ICD-10-A53.9) between January 2005 and December 2016 were retrieved. Cases lacking serological confirmation (Venereal Disease Research Laboratory – VDRL) or Fluorescent Treponemal Antibody Absorption (FTA-ABS) were excluded. The following data were collected from the medical records: age, sex, description of oral lesion, affected site, disease stage, serological tests, and treatment.

Data were organized into a database and descriptive analyses were conducted. The Pearson chi-square and *t*-test test were used to evaluate the association between the variables sex, age, site, clinical presentation, and stage. The level of significance was set at 5%. Analyses were performed using SPSS® version19.0 for Windows.

## Results

**Figure 1:**
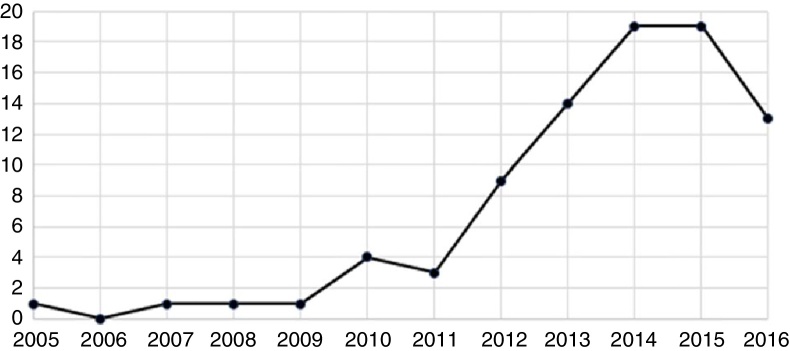
Number of cases diagnosed as acquired syphilis with oral manifestations between 2005 and 2016 at the Oral Medicine Service – HMOB.

A total of 85 diagnoses of acquired syphilis were registered from 2005 to 2016, with a marked increase in the number of diagnoses per year in the last 5 years (Fig. 1).

**Figure 2:**
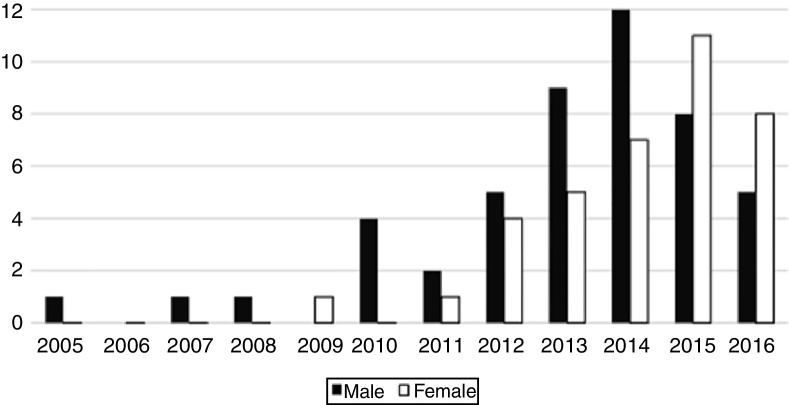
Annual frequency distribution of acquired syphilis with oral manifestations according to patient's sex.

**Figure 3:**
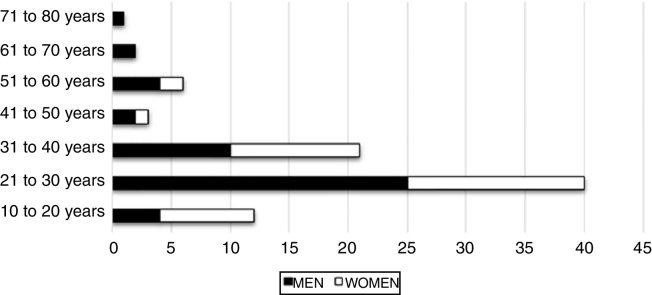
Frequency distribution of acquired syphilis with oral manifestations between 2005 and 2016 according to sex and age.

Forty-eight patients were males (56.5%), while 37 were females (43.5%). The traditional male predominance changed over the past 2 years, in which female patients have begun to outnumber male patients (Fig. 2). Patients ranged from 16 to 76 years of age, with a mean age of 29.58 years. Females had a lower mean age (26.46) than males (31.98) (*p* < 0.05) at diagnosis. Most patients were diagnosed in the third decade of life (40%–47%), followed by the fourth decade (21%–24.7%), however the number of women affected in the second decade (*n* = 14) was higher than men (*n* = 4) (*p* < 0.05). Therefore, the total of patients from 21 to 40 years of age was 61 cases, representing 71.7%. Ages below or above this range represented 24 cases (28.3%). Acquired syphilis was more frequent in men than in women in almost all age groups, except in the second and fourth decades of life (*p* < 0.05) (Fig. 3).

Most patients (72%–84.7%) lived in the city of Belo Horizonte (the state capital where the hospital is based), although 13 individuals (15.3%) were from neighboring cities.

Patients sought the Oral Medicine Service because of oral lesions. Some of them have reported painful symptoms or difficulty in swallowing. Most noticed the presence of the lesions two weeks before consultation.

**Figure 4:**
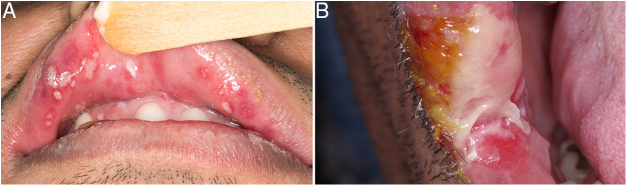
Oral syphilis represented by multiple (A) and single (B) ulcer and mucous plaque.

Oral lesions were mainly described as single (96.5%) ulcerations (78.8%). The most affected sites were the tongue and lips (23%–27.1% each) (Fig. 4). When the lesions were in multiple sites they were usually represented by mucosal plaques (*p* < 0.05). Comparisons between the other variables did not show statistical association.

A diagnosis of the secondary stage of syphilis was established in 94.1% of the cases, and all patients had tested positive for VDRL and FTA-ABS. All the 5 cases of primary syphilis were diagnosed in men (*p* < 0.05). Thirty patients (35.3%) underwent additional serological tests, such as blood count and anti-HIV. It is important to note that all of the 28 patients tested for anti-HIV presented a positive result.

Penicillin G benzathine (84.7%) and doxycycline (15.3%) were the drugs used for treatment. The summary of our study's results can be seen in Table 1.

**Table 1:** Clinical features of 85 patients diagnosed with acquired syphilis due to oral manifestations.

Variables	*n*	%
*Sex*		
Male	48	56.5
Female	37	43.5

*Age* (years-old)		
<20	12	14.2
21–30	40	47.0
31–40	21	24.7
41–60	9	10.6
61–80	3	3.5

*Number of oral lesions*		
Single	82	96.5
Multiple	3	3.53

*Site*		
Lips	23	27.1
Tongue	23	27.1
Cheek	14	16.5
Soft palate	8	9.4
Palatine tonsil	6	7.0
Oropharynx	6	7.0
Labial comissure	1	1.2
Mouth floor	1	1.2
Missing	3	3.5

*Clinical presentation*		
Ulcers	67	78.8
Mucosal plaque	17	20.0
Verrucous	1	1.2

*Syphilis stage*		
Secondary	80	94.1
Primary	5	5.9

*Treatment*		
Penicillin G benzathine	72	84.7
Doxycycline	13	15.3

## Discussion

Acquired syphilis is a sexually transmitted disease that develops through three stages. In the primary stage, the syphilitic complex is the main characteristic. This encompasses the “cancrum”, which appears at the inoculation site of *T. pallidum*, along with lymphadenopathy. No matter what the treatment, these initial lesions can resolve naturally, while *T. pallidum* further disseminates. The secondary stage is characterized by multiple mucous and cutaneous lesions, along with lymphadenopathy. Once again, these lesions can resolve spontaneously, and *T. pallidum* remains as a latent infection, however, about one third of all patients with untreated secondary syphilis develop the tertiary form.^2^

A patient's oral mucosa can be affected in all three stages, but it is most commonly affected in the secondary stage, which was confirmed in the present study. Secondary syphilitic oral lesions are usually multiple and more diversified than the single ulcer of the primary stage. Nevertheless, a single lesion may be the only manifestation that appears in the secondary stage. Oral lesions include mucous patches, macule, papule, and a nodular/ulcerative form. The presence of maculopapular skin lesions, lymphadenopathy, and medical history most often guide the clinician toward the proper diagnosis of secondary syphilis.^3^

Syphilitic lesions in the oral mucosa are known to be quite variable, simulating diverse diseases,^14^ such as HIV, lichen planus, eosinophilic ulcers, traumatic ulcers, lymphomas, leukoplakia, gonorrhea, squamous cell carcinoma, and necrotizing sialometaplasia. Accordingly, the clinical presentation found herein included ulcers, mucous plaques, and verrucous lesions. Since a histopathological routine exam is nonspecific, serological tests are essential to reaching the final diagnosis.

The serological tests used to diagnose syphilis are classified as nontreponemal and treponemal.^4^ The nontreponemal tests (VDRL and Rapid Plasma Regain – RPR) are nonspecific, although they are faster and cheaper,^6^ and are widely used for screening and disease detection. These tests are reactive in the secondary and latent phases and are less sensitive in patients with primary syphilis. VDRL and RPR may be reactive in other diseases, such as systemic lupus erythematosus, ulcerative colitis, and Rickettsial disease.^15^ The treponemal tests (FTA-ABS, *T. pallidum* Haemagglutination – TPHA), and Microhemagglutination Assay – MHA-TP) are more specific and sensitive, and thus become positive in the early stages of the disease. However, they are more expensive. Despite the limitations pointed out above, the serological tests, along with a well-conducted clinical examination, play a crucial role in the diagnosis of syphilis.^3^ All patients investigated in the current study presented a serological confirmation by VDRL and FTA-ABS.

The occurrence of syphilis used to be a great health problem before the discovery of penicillin.^16^ After the antibiotics era, syphilis remained a controlled infection for some decades. Nevertheless, a new burden is now being reported worldwide.^5^, ^8^ It is important to note that this resurgence of syphilis also coincides with a resurgence of HIV in society.^6^, ^10^

The present study confirmed this resurgence of syphilis, as can be seen in the alarming increase of the disease over the past five years. Social and behavioral aspects seem to play important roles in this scenario and have been pointed out as major reasons for this reemergence. A decrease in safe sex practices, the optimism with antiretroviral treatment, recreational drug use, and erectile dysfunction medication use are some examples of behavioral changes that incur higher risks.^6^

Cases were searched in the present study though a retrospective study based on the diagnosis of acquired syphilis (ICD-10-A53.9). All notified cases were of patients with oral lesions, stressing the importance of training oral health professionals in identification of acquired syphilis.

The present study identified the most affected age range to be from 20 to 39 years of age, which is exactly the reproductive age.^5^ Moreover, the increased frequency of the disease in female patients also opens the door to the risk for congenital syphilis. Therefore, it is pivotal to offer the population preventive and educational actions to avoid the vertical transmission of syphilis.^11^, ^14^ Patients above 60 years of age represented only 3.5%, while young people in their early 20s represented 14.2%. Although the most frequent cases are between 20 and 39 years of age, there are studies showing a rise in cases among the elderly (60–76 years of age).^7^, ^17^ This could be explained by the increase in the elderly population, the development of medicine to treat erectile dysfunction, as well as other possibilities of sexual contact.^18^ The involvement of these extremes in ages highlights the need for broader public health actions.

Reports in the literature show an important association between syphilis and HIV infection.^19^, ^20^ It is believed that syphilitic lesions represent a potential gateway to HIV, in turn facilitating infection. In addition, both diseases share the sexual route of infection.^10^, ^21^ Other studies have pointed out that it is essential for all patients suspected of being infected with syphilis to undergo HIV testing.^18^ In Brazil, the anti-HIV test has been recommended in cases of suspected sexually transmitted disease since 2016, following recommendation of the Federal Medical Council. This explains why only a few patients underwent this test. Likewise, in the present study, all patients screened for HIV presented a positive serology. Hence, informative actions for safe sex may be efficient in reducing syphilis, HIV, and other sexually transmitted diseases. Moreover, it is important that all sexual partners of syphilis patients be screened as well.

Penicillin remains the treatment of choice for syphilis,^22^, ^23^ and most patients were treated with penicillin G benzathine. Interestingly, a single or double dose protocol of azithromycin is also being advocated to improve treatment adherence.^24^, ^25^

Retrospective studies using medical records have the advantage of providing information at a low cost; however, data collected are dependent on how complete the information is. Moreover, in the present study, cases of acquired syphilis in patients not presenting oral lesions could not be diagnosed and the accurate numbers may be underestimated. Despite these limitations, the results described in this paper are in consonance with the literature.

## Conclusion

In conclusion, the present study corroborates with reports on the new syphilis burden in recent years. The study reported on 85 cases of acquired syphilis diagnosed through oral lesions, highlighting the need for the continuous education and training of oral health professionals. Patients of all ages and sexes were affected, reinforcing the need for more widespread preventive actions.

## Funding

This work was supported by FAPEMIG (State of Minas Gerais Research Foundation), 10.13039/501100002322CAPES (Coordination for the Improvement of Higher Level Education Personnel), and PRPq/UFMG grant number #05/2016 (Pró Reitoria de Pesquisa UFMG). Michelle Danielle Porto Matias is a FAPEMIG scholarship.

## Conflicts of interest

The authors declare no conflicts of interest.
